# Targeting RNA G‐quadruplexes as new treatment strategy for *C9orf72* ALS/FTD

**DOI:** 10.15252/emmm.201708572

**Published:** 2017-11-24

**Authors:** Martin H Schludi, Dieter Edbauer

**Affiliations:** ^1^ German Center for Neurodegenerative Diseases (DZNE) Munich Munich Germany; ^2^ Munich Cluster for Systems Neurology (SyNergy) Munich Germany

**Keywords:** Neuroscience, Pharmacology & Drug Discovery

## Abstract

The recent discovery of a pathogenic expansion of a (GGGGCC)_n_ repeat in the *C9orf72* gene in amyotrophic lateral sclerosis (ALS) and frontotemporal dementia (FTD) led to a burst of mechanistic discoveries. In this issue, Simone *et al* (2018) describe novel compounds targeting the G‐quadruplex (G‐Q) structure of the (GGGGCC)_n_ repeat RNA that alleviate the hallmarks of *C9orf72* disease in patient‐derived neurons and increase survival in a *Drosophila* model. Lack of overt off‐target effects and toxicity suggest that these small molecules are promising lead compounds to the development of a therapy.

Since the discovery of the (GGGGCC)_n_ repeat expansion upstream of the coding region of *C9orf72* as the most common genetic cause of ALS and FTD, tremendous progress toward understanding disease mechanisms and developing therapies has been made (Edbauer & Haass, 2016). The repeat RNA forms small foci within the nucleus and is thought to sequester several RNA‐binding proteins and thereby alter gene expression and splicing. Surprisingly, both sense and antisense transcripts are translated in all reading frames by an unconventional mechanism into five co‐aggregating dipeptide repeat (DPR) proteins: poly‐GA, poly‐GP, poly‐GR, poly‐PA, and poly‐PR. The DPRs also bind key cellular proteins (including RNA‐binding proteins), and their relative role in pathogenesis is under intense investigation. The so‐called repeat‐associated non‐ATG (RAN) translation has been first discovered for (CAG)_n_ expansion disorders (Zu *et al*, 2011) and was later reported for several other repeat expansions diseases (Cleary & Ranum, 2017). The mechanism remains elusive, but seems to depend on the secondary structure of the repeat RNA. Therefore, several groups have analyzed the RNA structure of (GGGGCC)_n_ repeats *in vitro* and *in vivo* and found that the repeat RNA can form both so‐called G‐quadruplexes (G‐Qs) and hairpins. G‐Qs are four‐stranded structures containing stacks of four guanines that associate through Hoogsteen hydrogen bonding within one plane (Fig 1). This structure can form from a single or up to four separate RNA or DNA strands. In contrast to G‐Qs formed from DNA, RNA‐based G‐Qs are more stable and compact as a consequence of more intramolecular interactions. Moreover, RNA G‐Qs preferentially assemble in a parallel conformation. Thus, DNA and RNA G‐Qs can be selectively targeted. Endogenous RNA G‐Qs within untranslated regions and introns regulate transcription, alternative splicing, and protein binding. Hairpins composed of a base‐paired stem and a loop are the most common RNA structures and can also affect transcription and alternative splicing. Thus, targeting the secondary structure of the disease‐associated (GGGGCC)_n_ RNA is a potential therapeutic strategy.

**Figure 1:**
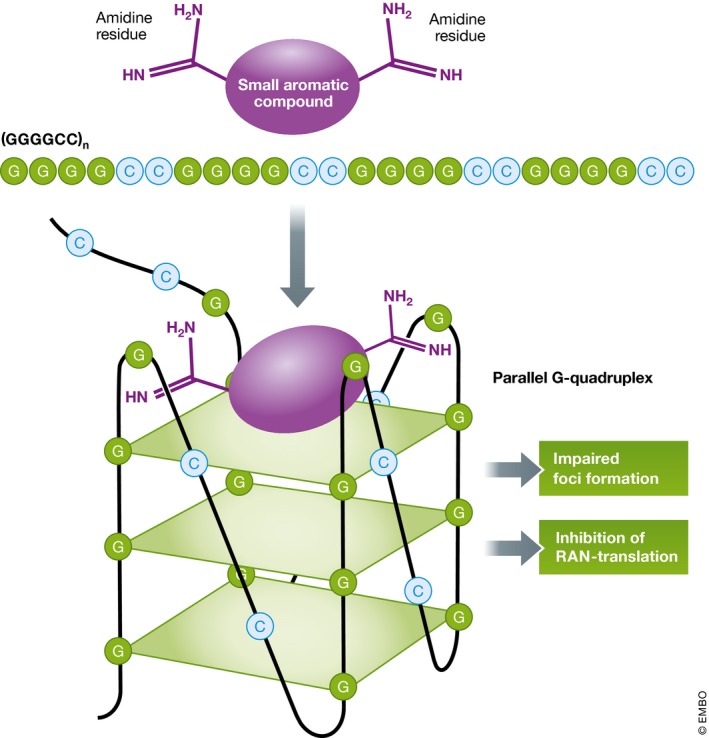
Small molecules stabilize the GGGGCC RNA G‐quadruplex structure Small aromatic compounds with two amidine residues preferentially interact with the parallel G‐Q of the (GGGGCC)_n_ repeat RNA and stabilize this structure. Stabilization of the G‐quadruplex structure reduces RNA foci formation and inhibits repeat translation. Note that other G‐Q confirmations are possible.

In 2014, Su *et al* repurposed a compound originally identified as an interactor of (CGG)_n_ in fragile X‐associated tremor ataxia syndrome for the *C9orf72* repeat RNA (Su *et al*, 2014). This study describes in total three compounds (*1a, 2, 3*), which interfere mainly with the hairpin structure of the (GGGGCC)_n_ RNA resulting in translational inhibition. In patient‐derived neurons, compound *1a* significantly reduced RNA foci and DPR proteins and showed no overt toxicity, but their more potent compound *2*, an aromatic diamidine, was too toxic to validate in patient‐derived neurons. None of the compounds were tested in animals.

In contrast, Simone *et al* (2018) specifically targeted the G‐Q structure and screened a library of 138 small molecules with known or suspected binding to G‐Q structures (Fig 1). They used a FRET‐based melting assay for the initial screen and selected three compounds that had a much larger effect on the G‐Q formation of (GGGGCC)_n_ RNA than of (GGGGCC)_n_ DNA. The three best compounds have a nearly identical atomic structure and are, like the most potent compound in Su *et al*, aromatic diamidines. Circular dichroism spectroscopy confirms direct binding to repeat RNA with 200–400 nM affinity. At 1 μM concentration, two of the compounds reduced RNA foci in iPSC‐derived spinal motor neurons and cortical neurons by about 50%. At higher concentration (16 μM) and later time points, both compounds also reduced poly‐GP expression by up to 50%, which is likely due to the long half‐life time of poly‐GP. In contrast to the aromatic diamidine compound from Su *et al*, no toxicity was observed at the effective concentration. In addition to these biochemical and cellular assays, Simone *et al* (2018) fed their best compound (DB1273) to flies modeling (GGGGCC)_n_ repeat toxicity (Mizielinska *et al*, 2014). Adult flies expressing (GGGGCC)_36_ showed a pronounced reduction in poly‐GP. Moreover, feeding larvae with the compound led to a modest increase in survival. Since poly‐GR is the main driver of toxicity in this model, Simone *et al* (2018) also developed a new poly‐GR immunoassay to directly show effect on the main toxic species. Indeed, stabilizing the G‐Q structure of (GGGGCC)_36_ using DB1273 also reduced poly‐GR levels by 33%, which is consistent with the moderate survival benefit. Since brain penetrance of DB1273 is still low, medicinal chemists may be able to improve the *in vivo* effects significantly in the future.

The Isaacs laboratory is covering a lot of ground already by validating their novel compounds in a fly model. The next obvious step would be treating mice expressing the (GGGGCC)_n_ repeat. One strategy to improve delivery to the CNS might be biopharmaceutical modifications such as incorporation into nanospheres or nanocapsules (Lu *et al*, 2014). Another way to boost the efficiency of the compounds could be *in situ* CLICK chemistry, because 1,3‐dipolar cycloaddition of alkynes and azides could promote cross‐linking of the modified compounds to (GGGGCC)_n_ G‐Qs *in vivo* (Di Antonio *et al*, 2012). The report from Simone *et al* (2018) also highlights a gradual shift in the drug discovery world. In 2017, targeting specific RNA molecules by small molecules is becoming increasingly feasible (Bernat & Disney, 2015), although antisense oligonucleotides still have superior efficacy. However, antisense oligonucleotides require invasive application and come with a hefty price tag (Jiang *et al*, 2016).

What do these new findings mean for our understanding of the pathological hallmarks of *C9orf72* disease? Apparently, stabilizing either the G‐Q or the hairpin structure can reduce RNA foci formation and inhibit translation of the (GGGGCC)_n_ repeat. However, it is unclear whether the compounds actually dissolve RNA foci or just impair their detection. It also remains elusive whether these compounds specifically inhibit RAN translation or would also inhibit ATG‐initiated translation of the structured repetitive RNA. RAN translation seems to highly depend on the RNA structure, because only (CAG)_n_ but not (CAA)_n_ repeats are translated into poly‐Q in the absence of an ATG‐start codon (Zu *et al*, 2011), suggesting that targeting the secondary structure of RAN‐translated repeat RNAs is a potential strategy to slow or stop disease progression. A compound affecting RAN translation of different repeat RNAs might be beneficial for many diseases (Cleary & Ranum, 2017).

In conclusion, this paper is an encouraging and timely study, because our understanding of *C9orf72* pathogenesis is growing rapidly and the first treatment options, such as antisense oligonucleotides are already on the horizon (Jiang *et al*, 2016). Patients can only benefit from the intense interest in the *C9orf72* mutation as academia and industry race for a treatment.
